# Editorial: Advances in understanding synaptic function and its dysfunction in neurological disorders

**DOI:** 10.3389/fnmol.2023.1239315

**Published:** 2023-06-29

**Authors:** Farhan Mohammad, Mohd. Farooq Shaikh, Yasir Ahmed Syed, Fadel Tissir

**Affiliations:** ^1^College of Health and Life Sciences, Hamad Bin Khalifa University, Education City, Qatar; ^2^School of Dentistry and Medical Sciences, Charles Sturt University, Orange, NSW, Australia; ^3^School of Biosciences, Neuroscience and Mental Health Innovation Institute and School of Biosciences, Cardiff University, Cardiff, United Kingdom

**Keywords:** synaptic plasticity, cognition, neurological disorder, animal models, molecular mechanism

Neurodevelopmental and psychiatric disorders, which currently lack effective treatment strategies (Aishworiya et al., 2022), pose a significant challenge to worldwide public health systems (Kularatna et al., 2022). One of the core clinical phenotype associated with these disabling disorders are cognitive deficits, which is likely due to molecular mechanisms that affect long-term changes in synaptic development, function and plasticity (Parenti et al., 2020). Understanding these mechanisms are the crucial first step for addressing cognitive impairments associated with these complex neurological conditions (Kozubski et al., 2021). Toward this purpose, our Research Topic focuses on the intricate aspects of brain cognitive functions, including the role of specific proteins, therapeutic interventions, modulation of neuronal circuits, and the impact of molecular mechanisms on cognitive impairments.

Animal models have played a crucial role in validating target genes (Salim et al., 2021), identifying potential interventions and therapies (Berry-Kravis et al., 2018; Muhle et al., 2022), modifying the progression of clinical phenotypes associated with neurological disorders (Díaz-Caneja et al., 2021), and mitigating cognitive decline (Whelan et al., 2022). The utilization of animal models, both mammalian and non-mammalian, has significantly contributed to our knowledge of synapse formation (Ruiz-Canada et al., 2004; Peters et al., 2017), function (Peters et al., 2017), and remodeling through learning (Li et al., 2009; Gatto and Broadie, 2011; Zhao and Bhattacharyya, 2018). Within this Research Topic, we delve into the intricate aspects of communication and information processing within neuronal circuits and systems. The included articles explore the roles of an atypical kinesin protein responsible for microtubule depolymerization and the intricate role of the K^+^-Cl^−^ cotransporter (KCC2) in regulating cognitive function in mice. These studies examine how changes in these proteins affect the structural organization, dynamics, and functionality of brain circuits. A study investigates the role of neural stem cell-derived extracellular vesicles and toxins from the venom in neuron regeneration and recovery from brain injuries. Additionally, the Research Topic highlights the significance of the Drosophila model system in examining and validating the roles of identified genes.

Ruiz-Reig et al. utilized a conditional knockout mice and demonstrate that specific depletion of KIF2A in GABAergic interneurons leads to abnormal behavior and increased susceptibility to epilepsy. KIF2A, an atypical kinesin protein with microtubule depolymerizing activity, is involved in the development and function of inhibitory cortical circuits. These findings provide valuable insights into how dysregulation of KIF2A contributes to alterations in neuronal circuits, imbalance in excitation/inhibition, and epilepsy. Furthermore, KIF2A is necessary for adult neurogenesis and the tangential migration of neuroblasts in the rostral migratory stream toward the olfactory bulbs. Thus, the study broadens our understanding of the crucial role of KIF2A neuronal circuits and its implications in neurodevelopmental disorders such as epilepsy.

While the brain has limited inherent regenerative capacity in comparison to other organs. However, ongoing research aims to uncover mechanisms and develop interventions to enhance brain plasticity and promote regeneration, holding potential for treating neurological disorders and promoting recovery from brain injuries. In this direction, Ocaña et al. revealed novel findings regarding the therapeutic potential of neural stem cell-derived extracellular vesicles (NSC-EVs) for nervous tissue damage and regeneration. The study demonstrated that NSC-EVs enhance NSC proliferation, promote neuronal differentiation, and restore the proliferative capacity of NSCs affected by oxidative stress. Additionally, NSC-EVs demonstrated the ability to ameliorate neuronal damage, improve neuronal plasticity, and enhance parameters associated with neuron function. These findings suggest that NSC-EV-based therapies hold promise for expanding neurogenesis and restoring neuronal plasticity in the treatment of nervous tissue damage.

In another study published within this Research Topic, Keimasi et al. purified and identified the omega-agatoxin-Aa2a protein from the venom of Agelena labyrinthica. The purified omega-agatoxin-Aa2a was found to interact with N-type voltage-gated calcium channels (VGCCs) and potentially block them, which is significant considering the role of calcium channels in neurotransmission. The administration of omega-agatoxin-Aa2a showed promising effects in controlling excitotoxicity, preserving synaptic markers, preventing neurodegeneration, and improving cognitive memory and learning performance in NMDA-induced models.

With a focus on understanding the modulation of hippocampal learning and memory by GABAergic transmission, Kreis et al. delve into the intricate role of the K^+^-Cl^−^ cotransporter KCC2 in regulating cognitive function in mice. This study investigated the tight regulation of intracellular chloride concentration ([Cl^−^]i) through cation-chloride transporters and revealed that a reduction in KCC2 expression led to an increase in [Cl^−^]i, reinforcing the spike responses of CA1 pyramidal neurons in the hippocampus to specific stimuli. Consequently, adult mice exhibited impairments in both spatial and non-spatial learning. These findings highlight the essential role of KCC2 in maintaining proper chloride equilibrium for synaptic plasticity and memory formation. It also demonstrated that the cognitive impairments resulting from reduced KCC2 expression could be largely mitigated by treatment with bumetanide, an antagonist targeting the Na^+^-K^+^-Cl^−^ cotransporter NKCC1 responsible for Cl^−^ import into neurons. This discovery emphasizes the significance of NKCC1 as a potential therapeutic target for cognitive disorders. The study's implications extend to the understanding of the underlying causes of neurological and psychiatric diseases, as alterations in NKCC1/KCC2 expression or activity may contribute to epilepsy, pain, schizophrenia, Alzheimer's disease, and autism. Overall, this study elucidates the role of chloride equilibrium in synaptic plasticity and memory formation. By identifying KCC2 and NKCC1 as potential targets, this study paves the way for the advancement of innovative therapeutic strategies for cognitive disorders.

Lastly, Liu et al. present their research on epilepsy candidate genes, namely Tango14, Klp3A, Cac, and Sbf, using the Drosophila model. This study demonstrates the optimized strategies employed by the researchers and validates the effectiveness of their screening system, which offers a viable alternative to rodent models. Given the vast number of genes and their variants identified through exome sequencing, the Drosophila model proves to be a valuable tool for confirming epilepsy candidate genes in a more efficient and feasible manner.

In summary, this Research Topic presents a series of articles which explores numerous aspects of cognitive functions, including the role of specific proteins, therapeutic interventions, modulation of neuronal circuits, and deepen the understanding of molecular mechanisms associated with cognitive impairments. These studies highlight the significance of preclinical models toward better understanding of synaptic plasticity and function. Furthermore, the series of articles in his Research Topic sheds light on the complex nature of brain cognitive functions and offers promising avenues for future research and therapeutic development in a clinically meaningful way.

## Author contributions

FM: conceptualization and writing original draft. FM, MS, FT, and YS: writing revision. All authors contributed to the article and approved the submitted version.
